# Driving forces for strengthening the surveillance of Chagas disease in the Brazilian Amazon by “training the eyes” of malaria microscopists

**DOI:** 10.1590/0037-8682-0423-2019

**Published:** 2020-03-16

**Authors:** Wuelton Marcelo Monteiro, Maria das Graças Vale Barbosa, Jorge Augusto de Oliveira Guerra, Gisely Cardoso de Melo, Layla Rowena Albuquerque Barbosa, Kim Vinicius Amaral Machado, Rebeca Linhares de Abreu, Marcus Vinicius Guimarães de Lacerda

**Affiliations:** 1 Gerência de malária da Fundação de Medicina Tropical Dr. Heitor Vieira Dourado, Manaus, AM, Brasil.; 2 Universidade do Estado do Amazonas, Programa de Pós-Graduação em Medicina Tropical, Manaus, AM, Brasil.; 3 Fundação Oswaldo Cruz, Instituto de Pesquisas Leônidas e Maria Deane, Manaus, AM, Brasil.

**Dear Editor:**

Surveillance activities that are well developed in one area may act as driving forces for strengthening surveillance activities in other areas^1^. For instance, in the vast Amazon region in which malaria transmission mostly occurs, often in remote areas, there are pockets of biodiversity with varied triatomine vectors and mammalian *Trypanosoma cruzi* reservoirs, and thus, there is a risk of Chagas disease transmission^2^. This overlap offers possible synergies and opportunities for the use of common resources for the surveillance of both of the aforementioned protozoan febrile diseases.

Hundreds of thousands of febrile patients are currently attended through a network of around 3,000 malaria diagnostic and treatment units across the Amazon^3^. Acute Chagas disease clinically appears as an undifferentiated febrile illness and, in the Amazon, it is often initially attributed to malaria due to the higher prevalence of malaria.

Since a distinctive characteristic of acute *T. cruzi* infection is the notable parasitemia in stained slide preparations, several cases of acute Chagas disease have been incidentally discovered in northern Brazil^4^. In recent decades, new acute cases of Chagas disease in the Amazon region have been sporadically reported in the literature, mostly as outbreaks^4^ ^,^ ^5^ ^,^ ^6^ ^,^ ^7^ ^,^ ^8^. In almost all Chagas disease outbreaks, cases were incidentally identified during investigations of acute febrile syndrome without apparent cause. Because of the frequent systematic diagnosis of malaria, *T. cruzi* trypomastigotes were found, highlighting this screening approach to identify infected individuals^9^.

**FIGURE 1:**
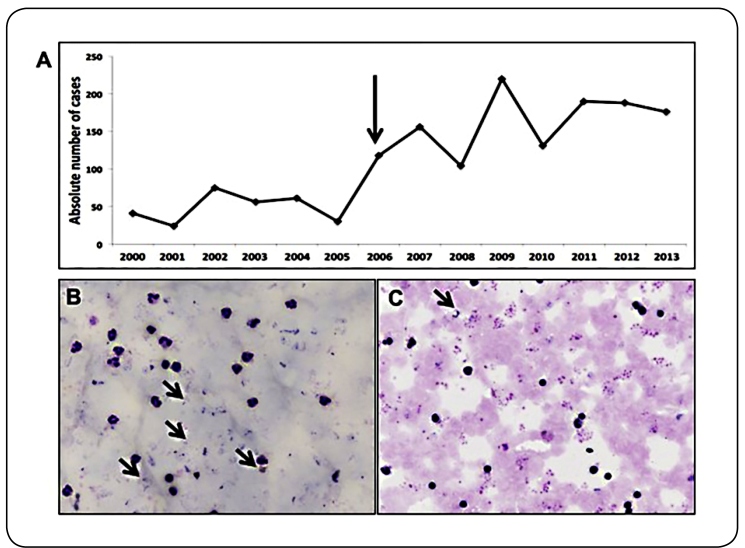
Historical series of acute Chagas disease in the Brazilian Amazon and parasites in thick blood smears. **(A)** An increasing trend of cases of acute Chagas disease in the Brazilian Amazon, especially after 2006, when the laboratory diagnosis and surveillance of this disease started to be performed together with malaria diagnostics. **(B)** Visualization of several parasitic forms of *Plasmodium vivax* in a thick blood smear, the method recommended by the Brazilian Ministry of Health. **(C)** A trypomastigote form of *Trypanosoma cruzi* in a febrile patient admitted for treatment at a malaria diagnostic center.

Chagas disease underdiagnosis in the past is a strong possibility, since previously there was no official recommendation to report suspected malaria cases in which thick blood smears showed trypanosomes. Moreover, malaria microscopists received no specific training in the identification and reporting of the presence of trypanosomes in blood slides. Historically, there has been a belief in the Amazon that Chagas disease is harmless, with mechanisms of transmission previously considered extraordinary or improbable since triatomines in this region have only wild habitats, never arriving at human dwellings^9^. However, with reduced incidence of Chagas disease in traditionally endemic areas following the implementation of large-scale vector control programs and screening of blood donors, changes in the epidemiology of acute Chagas disease are evidenced by the first outbreak officially investigated in Brazil in 2005, with probable oral transmission (Figure 1A)^9^. In one outbreak of acute Chagas disease, both patient blood and açai juice samples contained *T. cruzi* TcIV, indicating oral transmission^6^. This report underscored the important role of oral transmission in this disease. Epidemiologic investigations of most reported outbreaks in the Amazon region point to non-vectorial transmission, suggesting an increase in the number of cases of oral transmission, likely due to increased sensitivity of surveillance^6^.

From 2000 to 2013, an increased incidence of acute Chagas disease was observed (Figure 1A). In this period, 1,570 cases (112 cases/year) were reported in most Brazilian states, with 91% occurring in the states that are part of the Brazilian Amazon region. In 2009, the Brazilian Ministry of Health determined that microscopists should be trained by the State Central Public Health Laboratories to read thick blood smears not only for malaria detection but also for acute Chagas disease^10^ (Figures 1B and C).

The Brazilian Ministry of Health reported that after officially interfacing the malaria and acute Chagas disease surveillance systems, the mean number of cases of Chagas disease per year increased from 70.1 to 168.2. Additionally, decreased case fatality was observed, from 20% in 2005 to 1.1% in 2013^11^. This decrease likely occurred because microscopists were able to distinguish cases of Chagas disease from those of malaria, allowing earlier treatment and reducing the disease impact.

Today, new cases of both diseases occur almost exclusively in the Amazon region. Furthermore, as the number of diagnosed malaria cases decreases, the proportion of fever attributable to malaria drops. Consequently, if there is no change in routine surveillance, most of the remaining cases of acute febrile illness in the Amazon will be undiagnosed^12^. Therefore, new surveillance methods for endemic and emerging infectious agents and case management of febrile individuals will become a new priority^13^.
